# Exploration of the genetic neuroinflammatory environment in the human midcingulate cortex in Huntington’s disease

**DOI:** 10.1038/s43856-026-01526-5

**Published:** 2026-06-04

**Authors:** Mackenzie W. Ferguson, Shelley Scheepers, Thulani Palpagama, Malvindar Singh-Bains, Aodán Laighneach, Derek W. Morris, Clinton Turner, Lynette Tippett, Henry J. Waldvogel, Richard L. M. Faull, Andrea Kwakowsky

**Affiliations:** 1 Centre for Brain Research and Department of Anatomy and Medical Imaging, Faculty of Medical and Health Sciences, University of Auckland, Auckland, New Zealand; 2 Pharmacology and Therapeutics, School of Pharmacy and Medical Sciences, Institute for Health Discovery and Innovation, Institute for Clinical Trials, Galway Neuroscience Centre, University of Galway, Galway, Ireland; 3 Centre for Neuroimaging, Cognition and Genomics (NICOG), School of Biological and Chemical Sciences, University of Galway, Galway, Ireland; 4 Department of Anatomical Pathology, LabPlus, Auckland City Hospital, Auckland, New Zealand; 5 Centre for Brain Research and School of Psychology, Faculty of Sciences, University of Auckland, Auckland, New Zealand

**Keywords:** Huntington's disease, Chronic inflammation, Huntington's disease

## Abstract

**Background:**

Despite progress, the pathophysiology involving neuroinflammation in Huntington’s disease remains uncertain, and the genetic environment of the midcingulate cortex in the disease has not been investigated.

**Methods:**

Utilizing 14 Huntington’s disease cases (6 females and 8 males; age range 41-72) split into mood, motor and mixed symptomatology and nine control cases (3 females and 6 males; age range 53-72), we used mRNA sequencing to examine the midcingulate cortex transcriptome in Huntington’s disease and NanoString analysis to validate the differentially expressed transcripts. These genes underwent bioanalysis, including gene ontology enrichment, protein-protein interaction and cell-type enrichment analysis.

**Results:**

Here we show that multiple neuroinflammatory transcripts are overexpressed in the Huntington’s disease midcingulate cortex, such as those linked to classical complement, toll-like receptor signaling and AQP4 activity. However, related processes, such as chemokine activity, are downregulated, implying that a complex combination of gain and loss of neuroinflammatory function is occurring.

**Conclusions:**

In summary, neuroinflammation-related transcripts are overrepresented in Huntington’s disease cases with motor symptoms compared to mood and mixed. These findings suggest a potentially unique role for the midcingulate cortex in motor-specific neuroinflammatory pathophysiology.

## Plain language summary

Huntington’s disease (HD) is an inherited disease that causes the progressive breakdown of nerve cells in the brain. HD has a broad impact on a person’s functional abilities and results in mood, movement, thinking, and psychiatric problems. The midcingulate cortex (MCC) is a brain region that is impacted by HD pathology. Our project examined whether the degree of the immune system’s response, called inflammation, in the MCC correlates with the type of symptoms. We demonstrate that neuroinflammation-related gene products are increased in HD cases with motor symptoms compared to those with mood and mixed symptoms. These findings suggest a potentially unique role for the MCC in motor-specific neuroinflammatory pathology.

## Abstract (web summary)

Ferguson et al. analyze transcriptomic changes in the midcingulate cortex from Huntington’s disease (HD) brain samples. Authors find that neuroinflammation-related transcripts are overrepresented in HD cases with motor symptoms suggesting a potentially unique role for this brain region in motor-specific neuroinflammatory pathophysiology.

## Introduction

Huntington’s disease (HD) is an autosomal dominant neurodegenerative disorder caused by expanded CAG trinucleotide repeats within exon one of the huntingtin gene^1^. This mutation leads to misfolded mutant huntingtin (mHTT) protein and neuropathology, including progressive neuronal death, striatal atrophy, and cortical thinning^2,3^. HD symptomatology involves motor symptoms, such as chorea, dystonia, bradykinesia, and rigidity, mood symptoms, including depression and apathy, and cognitive decline^4,5^.

Neuroinflammation is thought to contribute to the pathophysiology of multiple neurodegenerative disorders, including HD^6,7^. Neuronal stress or insults trigger multiple signaling pathways that provoke glia to transition into activated states and undergo gliosis^8,9^. These processes induce mechanisms that increase cytokine, reactive oxygen species, and prostaglandin production^10^. In diseased states, this facilitates neuronal stress, blood-brain barrier dysfunction, and eventually neuronal death, propagating a chronic, toxic cycle^11^. However, neuroinflammation is protective in normal physiology, and there is still some debate about whether neuroinflammation may hold a protective role in diseased states^12^. Importantly, the definitive role and consequences of neuroinflammation in HD are unclear^9^.

The middle cingulate cortex (MCC) is located medially within the cerebral cortex, above the corpus callosum^13^. The MCC has multiple roles in mood function and regulation, with projections to the limbic system and frontal cortex^13^. HD cases with predominant mood symptomatology show atrophy within the anterior cingulate cortex (ACC)^14^. It is unclear whether ACC atrophy extends to the MCC, although it is possible given the MCC’s functions and projections^14^. We recently reported that microglia in the MCC do not proliferate, but transition into reactive forms alongside increased mHTT burden, suggesting neuroinflammatory changes occur in HD in this brain region^15^. This study aimed to characterize inflammation-related differential gene expression in the MCC to reveal more about the neuroinflammatory environment in HD, aiding future therapeutic target identification. Neuroinflammatory markers, such as pro-inflammatory cytokines, are not differentially expressed, and there is no significant cell loss in the HD MCC. However, we demonstrate that neuroinflammation-related transcripts, such as those related to classical complement and toll-like receptor (TLR) signaling, are overrepresented in HD cases with motor symptoms compared to those with mood and mixed symptoms.

## Materials and methods

All post-mortem human MCC tissue was obtained from the Neurological Foundation Human Brain Bank at the Centre for Brain Research in Auckland, New Zealand. Donors were consented through a bequest process approved by the University of Auckland Human Participants Ethics Committee (approval: 011654) in accordance with the Declaration of Helsinki.

### Case preparation and evaluation

Brain perfusion and tissue preparation protocols align with those previously described within our laboratory^16^. Blocks containing the MCC were dissected from the left hemisphere and freshly frozen at −80 °C. These blocks were located dorsally to the corpus callosum and posteriorly to the ACC. A neuropathologist examined basal ganglia blocks from each case and assigned a pathological striatal grade between zero and four, as per the Vonsattel grading criteria^17^. The control cases had no neuropathological abnormalities on pathological examination. Tissue from each case was sectioned at 60 μm thickness onto slides using a cryostat. Six gray matter samples were collected per case, stored in RNase-free tubes at −80 °C, and homogenized prior to use in experiments. This study included 14 HD cases and nine control cases, with an average age of 59.0 and a post-mortem delay of 13.6 h. Control cases had no neurological disorders or psychiatric illnesses within their clinical notes. The HD cases were categorized into motor (*n* = 4), mood (*n* = 5), and mixed (*n* = 5) symptom profiles. Clinical information was obtained using a questionnaire and semi-structured interview, after which the data were reviewed and categorized into symptom profiles by two psychologists blinded to the anatomical information, as previously described^18^. Motor individuals presented with motor symptoms and no significant presentation of mood symptoms; mood individuals presented with predominantly mood disturbances during the progression of the disease, and mixed motor-mood individuals had significant levels of both motor and mood symptoms present during the progression of the disease, as reported in a previous study^14^.

### RNA extraction and library construction

Total RNA was isolated from three 60 μm HD MCC gray matter sections per case using the RNeasy® Lipid Tissue Mini Kit (74804, QIAGEN, Hilden, Germany), as per the manufacturer’s protocol. A NanoDrop spectrophotometer (117127, Thermo Fisher, Wilmington, NC, USA) obtained RNA concentration and quality measurements. RNA integrity (RIN) values were assessed using the RNA Nano 6000 Assay Kit of the Agilent 2100 Bioanalyzer (G2947CA, Agilent Technologies, Waldbronn, Germany), with the minimum RIN value obtained being 5.0, maximum being 8.4, and mean being 6.9. Library construction and mRNA sequencing were outsourced to Custom Science Ltd in Auckland, New Zealand. Sample mRNA was poly-T selected, chemically fragmented, and cDNA was synthesized, enabled by random hexamer priming. NEBNext adapters were ligated to the adenylated cDNA fragment 3′ ends, which was purified to isolate fragments 250–300 base pairs in length. The adapters’ hairpin loop was removed, and polymerase chain reaction (PCR) was performed to attain the final mRNA library. Qubit 2.0 was used for initial quantification, and Agilent 2100 measured the library insert size. Q-PCR was performed to quantify the mRNA library concentration (with an acceptable library quality being >2 nM).

### RNA sequencing

Fragment cluster generation and bridge amplification were undertaken using the Illumina TruSeq PE Cluster Kit v3-cBot-HS on a cBot Cluster Generation System, according to the manufacturer’s instructions. Next-generation sequencing was performed using Illumina’s HiSeq 2500 Ultra-High-Throughput Sequencing System (SY-401-2501, Illumina, San Diego, CA, USA), generating at least 50 million 150-base pair paired-end reads per sample. Raw sequence and quality information were filtered and processed into FASTQ files using in-house Perl scripts with Trimmomatic. Reads were aligned to the reference genome using Hisat2 version 2.0.5.

### RNA sequencing bioinformatics analysis

Read counts mapped to each gene were calculated using the union model in HTSeq-count (version 0.6.0). Differential gene expression analysis between all HD (and HD symptom groups) and control cases was performed on standardized read counts using the DESeq2 R package software (version 1.30.0) based on a negative binomial distribution. Heatmaps were created to compare differential gene expression from all HD, HD symptom profile, and control cases. Fragment per kilobase of transcript per million mapped reads (FPKM) values were used for hierarchical clustering analysis, and differential expression was expressed as *Z*-scores. The collated expression levels of all the samples were submitted to the R package pheatmap for bidirectional gene and sample clustering. Differential expression among samples is displayed as red being more upregulated and blue being more downregulated. Gene ontology (GO) enrichment analysis of differentially expressed genes was implemented by the topGO R Package (version 2.36.0; http://www.bioconductor.org). Human GO annotation information was downloaded from Ensembl’s Biomart database. GO terms with corrected *p* values of less than 0.05 were considered significantly enriched.

### Validation of RNA sequencing with NanoString nCounter analysis

The RNA sequencing results were validated using the NanoString nCounter gene expression analysis system. In total, 46 inflammation-related genes were selected for nCounter analysis. Nanostring Technologies supplied the oligonucleotide probe designs to target all transcripts for the selected genes. Six housekeeping genes were added to the nCounter Elements TagSet for data normalization (*ACTB, B2M, G6PD, POLR1B, GADPH*, and *TOP1)*. The RNA samples were diluted in RNase-free water to a total RNA concentration of 40 ng/µl. The nCounter assay was run on a 12-assay nCounter master kit (NanoString Technologies, 100052, Seattle, WA, USA) and nCounter Analysis system (NanoString Technologies, NCT-SYS-120, Seattle, WA, USA) according to the manufacturer’s instructions at the University of Auckland’s Grafton Clinical Genomics facility. The nSolver analysis software (version 4.0; NanoString Technologies) analyzed reporter probe counts and normalized the raw expression data using the housekeeping genes and positive controls. Background noise was removed by thresholding to negative controls. The mRNA expression data were further analyzed and graphed using Prism software (version 7; GraphPad Software).

### Protein–protein interaction analysis

Functional protein-association networks were created from our RNA sequencing data using the Search Tool for the Retrieval of Interacting Genes/Proteins (STRING) database (version 12.0; https://string-db.org). STRING accumulates physical and functional protein–protein interaction (PPI) data from genomic context predictions, high-throughput experimental data, conserved co-expression, text-mining, and curated knowledge in other databases^19^. These interactions are given a confidence score by integrating the probabilities from available evidence, with one being the highest confidence score^20^. We filtered novel transcripts and genes not recognized by STRING out of the inflammation-related genes inputted into STRING—leaving 19 upregulated and seven downregulated genes inputted for all HD cases.

### Expression-weighted cell-type enrichment analysis

Expression-weighted cell-type enrichment (EWCE) analysis was completed using the significantly differentially expressed genes found in whole genome RNA-sequencing results from the HD MCC (*n* = 223)^21^. EWCE analysis determines whether an inputted gene set is more highly expressed within certain cell types than can be expected from chance alone. We used single-cell RNA-sequencing data of the mouse nervous system from Zeisel et al. to determine the cell-type expression profiles used in this analysis^22^. There were 265 total cell types found in this study, including central and peripheral neurons, glia, immune cells, and vascular cells. EWCE was run using the “EWCE” R package (version 1.12.0), and brain-expressed genes (*n* = 15331) from the Human Protein Atlas were used as a background gene set. The analysis was bootstrapped 100,000 times, producing *p* values derived from the gene set’s performance against 100,000 random gene sets of the same size. After filtering, 82 gene hits were considered of the 223 total in all HD cases, 99 gene hits were considered of the 193 total in HD motor cases, 11 gene hits were considered of the 29 total in HD mood cases, and nine gene hits were considered of the 20 total in mixed HD cases.

### Immunohistochemistry and stereological cell quantification methods

Human brain tissue was processed by the Neurological Foundation Human Brain Bank as previously described. Blocks from the MCC, classified as CG2 in accordance with Waldvogel et al., for stereological cell quantification, were serially sectioned at 50 μm^16^. Every 10th section throughout the block was immunolabelled utilizing a free-floating 3,3′-diaminobenzidine (DAB)-peroxidase immunohistochemistry protocol in accordance with Waldvogel et al.^16^. The primary antibody utilized in this study was rabbit Neuronal Nuclei (NeuN), targeting nuclear protein expressed in neurons (1:1000, Millipore, ABN78, Lot 2972808, 3133550, and 3154915). Unbiased stereological quantification methods were utilized to determine the number of NeuN-positive neurons. The optical fractionator sampling probe was utilized for sampling through the StereoInvestigator software (Version 10, MBF Biosciences, MicroBrightField Ltd, USA). Cell number estimates were made using the probe in accordance with the following formula: *N* = ∑*Q*- × t/h × 1/asf × 1/ssf (where *N* = total number of neurons; ∑*Q*- = total number of neurons counted; *t* = average thickness of tissue sections; *h* = height of the dissector; asf = area sampling fraction; ssf = section sampling fraction). The coefficient of error (CE) was evaluated for the optical fractionator-derived cell estimates for each case. CE evaluation determined that the counting parameters employed in this study resulted in CE values of less than 0.1 for all cases counted.

### Statistics and reproducibility

RNA sequencing data *p* values were obtained using the two-tailed Wald test and adjusted for false discovery rate. Genes with an adjusted *p* value < 0.05 and log2 fold change ≥1 or ≤−1 were considered differentially expressed. Statistical analysis of Nanostring and stereological cell quantification data was performed using a two-tailed Mann–Whitney test for HD vs control, and a right-tailed Kruskal–Wallis test to compare the control vs HD symptom groups. Statistical significance was set at *p* ≤ 0.05%. Statistical analysis for stereological estimates utilized GraphPad Prism software (Version 8.0, GraphPad Software Inc., San Diego, CA, USA).

## Results

We conducted whole transcriptome Next-Generation Sequencing in HD symptom profile cohorts, including mood, motor, and mixed cases, utilizing subsequent bioanalysis to assess differentially-expressed inflammation-related mRNA transcripts. We used GO enrichment analysis and PPI analysis to determine if neuroinflammatory pathways are altered in the HD MCC.

### Inflammation-related differential gene expression in the Huntington’s disease midcingulate cortex and GO analysis

RNA sequencing analysis of 14 HD cases showed that 223 genes were differentially expressed with significantly adjusted *p* values, with 119 genes upregulated and 104 downregulated. Of these, 37 inflammation-related genes were differentially expressed, with 24 upregulated (Table 1) and 13 downregulated (Table 2). These 37 genes were selected to undergo NanoString nCounter analysis, of which 36 were differentially expressed, 24 were upregulated (Table 1), and 12 were downregulated (Table 2).

**Table 1:** Inflammation-related differential gene expression in all Huntington’s disease cases in mRNA sequencing and Nanostring analyses: upregulated genes

All HD significantly upregulated inflammation-related genes					
Gene name	Description	RNA sequencing		Nanostring	
		Adj *P* value	Log2 fold change	Adj *P* value	Log2 fold change
CHI3L1	Chitinase 3-like 1	<0.0001***	1.5664	<0.0001***	1.5967
SERPINA5	Serpin family A member 5	<0.0001***	3.2871	0.0017**	1.5725
P2RX7	Purinergic receptor P2X 7	0.0020**	1.1555	0.0191*	0.6342
ALOX5AP	Arachidonate 5-lipoxygenase activating protein	0.0030**	2.0391	0.0034*	1.1722
TMIGD3	Transmembrane and immunoglobulin domain-containing 3	0.0032**	1.3134	0.0002***	0.9516
SIGLEC8	Sialic acid binding Ig like lectin 8	0.0061**	1.3655	0.0006***	0.7382
SPP1	Secreted phosphoprotein 1	0.0062**	1.8910	0.0012**	1.2884
NGFR	Nerve growth factor receptor	0.0064**	2.2124	0.0043**	1.1363
SERPINA3	Serpin family A member 3	0.0245*	2.6233	0.0043**	2.4982
FKBP5	FK506 binding protein 5	0.0334*	1.3910	0.0016**	1.1410
SERPINA1	Serpin family A member 1	0.0336*	2.2038	0.0158*	0.9654
S100A9	S100 calcium-binding protein A9	0.0341*	2.8435	0.0012**	2.3058
TLR2	Toll-like receptor 2	0.0408*	1.3221	0.0043**	1.0977
C3	Complement C3	0.0434*	1.4408	0.0239*	0.7227
EVI2B	Ecotropic viral integration site 2B	0.0472*	1.1402	0.0130*	0.6987
AQP4	Aquaporin 4	0.0479*	1.0935	0.0086**	0.5669
CD44	CD44 molecule	0.0491*	2.0440	0.0043**	1.4961
C1QB	Complement C1q B chain	0.0498*	1.5072	0.0020**	1.0573
TLR7	Toll-like receptor 7	0.0507*	1.4462	0.0191*	0.6373
*Statistical significance only in RNA sequencing*		*Adj P value*		*Log2 fold change*	
C4B	Complement C4B	<0.0001***		2.5180	
C3AR1	Complement 3a Receptor 1	0.0335*		1.4258	
SLC5A11	Solute carrier family 5 member 11	0.0450*		1.4671	
TLR8	Toll-like receptor 8	0.0515*		2.6382	
C1QA	Complement 1 QA	0.0527*		1.4326	
*Statistical significance only in Nanostring*		*Adj P value*		*Log2 fold change*	
IL17RB	Interleukin 17 Receptor B	0.0130*		0.6559	
GJC2	Gap junction protein gamma 2	0.0275*		0.4846	
RELA	RELA proto-oncogene, NF-kB subunit	0.0191*		0.4092	
RNASE2	Ribonuclease A family member 2	0.0456*		0.9363	
STAT3	Signal transducer and activator of transcription 3	0.0456*		0.2303	

RNA sequencing—two-tailed Wald test; Nanostring—two-tailed Mann–Whitney test for HD vs control, and a right-tailed Kruskal–Wallis test to compare the control vs HD symptom groups. * *p* ≤ 0.05; ** *p* ≤ 0.01; *** *p* ≤ 0.001.

**Table 2:** Inflammation-related differential gene expression in all Huntington’s disease cases in mRNA sequencing and Nanostring analyses: downregulated genes

All HD significantly downregulated inflammation-related genes					
Gene name	Description	RNA sequencing		Nanostring	
		Adj *P* value	Log2 fold change	Adj *P* value	Log2 fold change
CCL8	C-C motif chemokine ligand 8	0.0003***	−4.8444	0.0387*	−0.7028
GPR85	G protein-coupled receptor 85	0.0004***	−1.0217	0.0002***	−0.4565
CCL4L2	C-C motif chemokine ligand 4 like 2	0.0013**	2.1080	0.0020**	−1.4440
CCL4	C-C motif chemokine ligand 4	0.0023**	−3.0228	0.0027**	−1.3389
CD8B2	CD8b2 molecule	0.0054**	−1.8274	0.0055**	−1.0910
GRP	Gastrin-releasing peptide	0.0124*	−1.3091	0.0069**	−0.8507
TNFSF10	TNF superfamily member 10	0.0131*	−1.8210	0.0012**	−1.2938
CXCL12	C-X-C motif chemokine ligand 12	0.0232*	−1.0596	0.0387*	−0.4970
CCL3L1	C-C motif chemokine ligand 3-like 1	0.0498*	−1.7448	0.0016**	−1.9614
*Statistical significance only in RNA sequencing*		*Adj P value*		*Log2 fold change*	
CD244	CD244 molecule	0.0261*		−1.6239	
UTS2B	Urotensin 2B	0.0341*		−1.2192	
MTRNR2L12	MT-RNR2 like 12	0.0449*		−1.8039	
SEMA3G	Semaphorin 3G	0.0488*		−1.2761	
Statistical significance only in Nanostring		*Adj P value*		*Log2 fold change*	
EGR1	Early growth response 1	0.0003***		−0.8960	
EGR2	Early growth receptor 2	0.0134*		−0.5485	
TDGF1	Teratocarcinoma-derived growth factor 1	0.0317*		−1.1800	

RNA sequencing—two-tailed Wald test; Nanostring—two-tailed Mann–Whitney test for HD vs control, and a right-tailed Kruskal–Wallis test to compare the control vs HD symptom groups. * *p* ≤ 0.05; ** *p* ≤ 0.01; *** *p* ≤ 0.001.

A range of inflammation-related genes were upregulated across all HD cases in mRNA sequencing and NanoString nCounter analyses (Table 1). Genes encoding TLRs, including TLR2 and TLR7, complement cascade proteins C1QB and C3, serpin family genes SERPINA1, SERPINA3, and SERPINA5, leukocyte marker CD44, and water-channel protein AQP4 were upregulated in both techniques. Additional genes were upregulated in the mRNA sequencing analysis only, such as the complement genes C4B, C1QA, and C3AR1, and TLR TLR8. RELA, an NF-$\kappa$B subunit, and STAT3 were upregulated in the Nanostring analysis, but not in mRNA sequencing. Additional inflammation-related genes were downregulated across all HD cases in both techniques, including chemokine encoding genes CCL4L2, CCL4, CCL8, CCL3L1, and CXCL12. Natural killer cell marker CD244, along with UTS2B, MTRNR2L12, and SEMA3G, was downregulated in the RNA sequencing results only. Conversely, in the Nanostring analysis only, EGR2 and TDGF1 were downregulated.

**Fig. 1:**
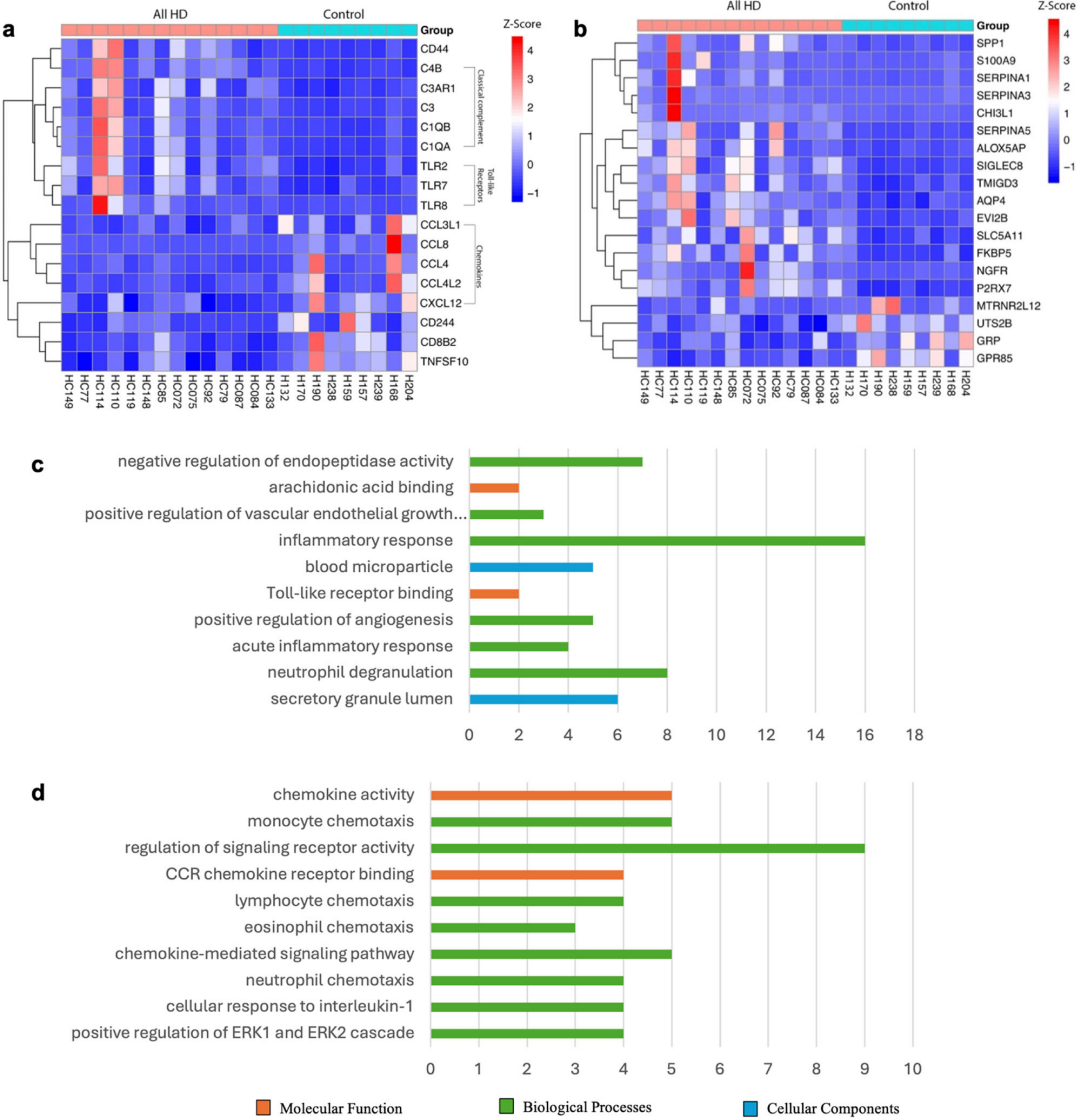
Heatmap and gene ontology analysis for all Huntington’s disease cases compared to control cases. **a** General inflammation-related genes with well-established roles in the literature. **b** Other inflammation-related genes expressed in the heatmap. **c** Top 10 upregulated inflammation-related gene ontology terms in all HD cases plotted against the number of significantly enriched genes expressed under these terms. **d** Top 10 downregulated inflammation-related gene ontology terms in all HD cases plotted against the number of significantly de-enriched genes expressed under these terms.

Heatmaps demonstrating inflammation-related differential gene expression in the mRNA sequencing analysis and the top 10 upregulated and downregulated inflammation-related GO terms across all HD cases demonstrate the cellular components, molecular functions, and biological processes associated with these genes (Fig. 1a, b). The four upregulated inflammation-related GO terms with significant adjusted *p* values (*p* ≤ 0.05) had multiple associated functions. However, the nine significantly downregulated biological process GO terms were clearly linked to chemotaxis, signaling pathway regulation, and cellular response to interleukin (Fig. 1c). Likewise, the two significantly downregulated molecular function GO terms were linked to chemokine activity binding (Fig. 1d).

### Protein–protein interaction analysis

**Fig. 2:**
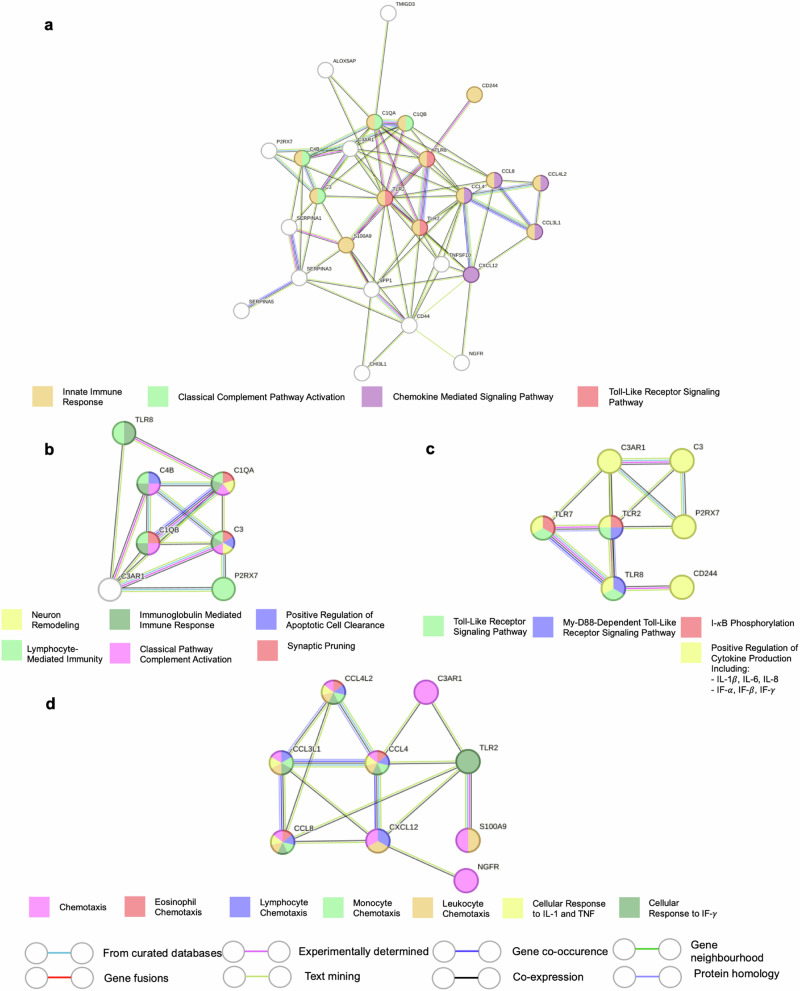
Protein–protein interaction analysis for inflammation-related differentially expressed genes in the midcingulate cortex in all Huntington’s disease cases. **a** Protein–protein interaction analysis for all inflammation-related differentially expressed genes in the HD MCC. **b** Protein–protein interaction analysis for classical complement and associated genes. **c** Protein–protein interaction analysis for toll-like receptor and associated genes. **d** Protein–protein interaction analysis for chemokine and associated genes.

The inflammation-related PPI analysis across all HD cases included 26 genes with 81 interactions, forming distinct clusters of TLR, complement, and chemokine-related interactions (Fig. 2). The HD motor cohort had the most protein interactions within the symptom groups, with 17 genes involved and 32 interactions. Conversely, the HD mood cohort showed no interactions between the inputted differentially expressed genes, and the mixed cohort only had six genes involved and five protein interactions (Supplementary Fig. 1).

### Cell-type enrichments of Huntington’s disease gene sets in the midcingulate cortex

EWCE analysis of the differentially expressed genes in all HD cases produced four significantly enriched cell types—two of which were activated microglia (MGL2, MGL3). The other two were cortical excitatory neurons (TEGLU17), and arterial vascular endothelial cells (VECA) (Supplementary Table 8). While mood and mixed cohorts did not produce any enriched cell types, the HD motor differential gene set produced 11. Five of these were varying types of oligodendrocytes (MFOL1, MFOL2, NFOL1, NFOL2, MOL2). TEGLU17 was also enriched in the motor cohort, alongside SCGLU10 (spinal cord excitatory neurons). Interestingly, two inhibitory neuron groups were also enriched (SCINH5 and MEINH5). Other enriched cell types included enteric glia (ENTG3) and hypothalamic peptidergic neurons (HYPEP2) (Supplementary Table 3).

### Inflammation-related differential gene expression in Huntington’s disease mood cases

HD mood cases had 29 significantly differentially expressed genes overall in the MCC (Table 3). Of these, 18 were upregulated, and 11 were downregulated. Seven inflammation-related genes were upregulated in HD mood cases within the RNA sequencing results. Of these, three genes were significantly upregulated in both the RNA sequencing and Nanostring datasets—CHI3L1, TMIGD3 and SERPINA5. The other four genes significantly upregulated in the RNA sequencing results only included ALOX5AP, SIGLEC8, C4B, and IL17RB. No significantly downregulated genes were found in the RNA sequencing results, but two significantly downregulated genes, CCL3L1 and TNFSF10, were identified in the Nanostring analysis (Table 3). No GO terms were significantly differentially expressed in the HD mood cohort, but four terms approached significance—“arachidonate 5- lipoxygenase activity,” “arachidonic acid binding,” “leukotriene-C4 synthase activity,” and “interleukin-17 receptor activity” (*p* = 0.0592).

**Table 3:** Inflammation-related differential gene expression in Huntington’s disease mood cases in mRNA sequencing and Nanostring analyses

Mood HD significantly upregulated inflammation-related genes					
Gene name	Description	RNA sequencing		Nanostring	
		Adj *P* value	Log2 fold change	Adj *P* value	Log2 fold change
CHI3L1	Chitinase 3-like 1	0.0041**	1.7993	0.0158*	1.4284
TMIGD3	Transmembrane and immunoglobulin domain-containing 3	0.0178*	1.3610	0.0335*	0.9812
SERPINA5	Serpin family A member 5	0.0163*	3.3929	0.0366*	1.7333
*Statistical significance only in RNA sequencing*		Adj *P* value		Log2 fold change	
ALOX5AP	Arachidonate 5-lipoxygenase activating protein	0.0178*		2.1357	
SIGLEC8	Sialic acid binding Ig like lectin 8	0.0192*		1.2017	
C4B	Complement 4B	0.0454*		1.8771	
IL17RB	Interleukin 17 Receptor B	0.0486*		1.1095	
**Mood HD significantly downregulated inflammation-related genes**					
*Statistical significance only in Nanostring*		Adj *P* value		Log2 fold change	
CCL3L1	C-C motif chemokine ligand 3-like 1	0.0409*		−2.1324	
TNFSF10	TNF superfamily member 10	0.0504*		−1.3579	

RNA sequencing—two-tailed Wald test; Nanostring—two-tailed Mann–Whitney test for HD vs control, and a right-tailed Kruskal–Wallis test to compare the control vs HD symptom groups. * *p* ≤ 0.05; ** *p* ≤ 0.01.

### Inflammation-related differential gene expression in Huntington’s disease motor cases

**Fig. 3:**
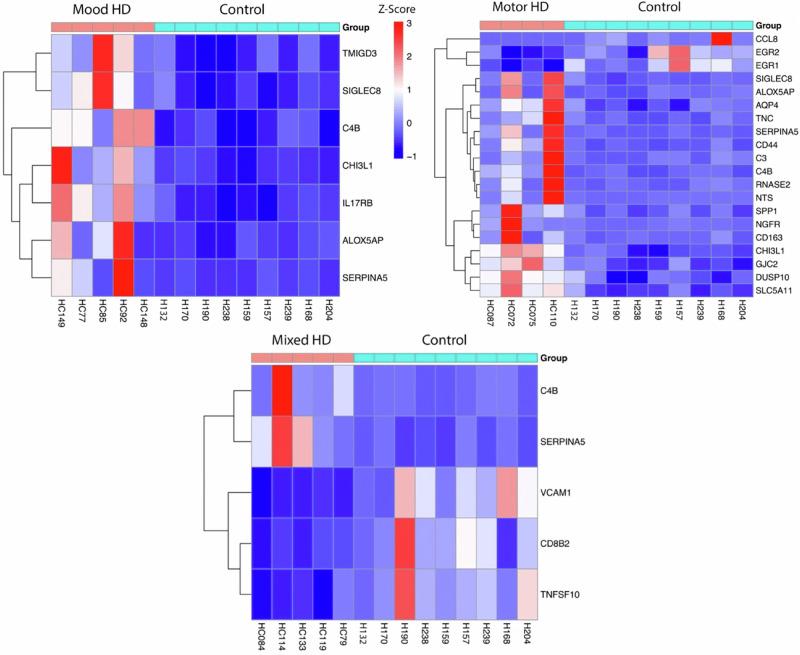
Heatmaps displaying inflammation-related differential gene expression in Huntington’s disease mood, motor, and mixed cases in mRNA sequencing.

HD motor cases had 193 significantly differentially expressed genes in the mRNA sequencing results in the MCC. Of these genes, 138 were upregulated, and 55 were downregulated. Seven inflammation-related genes were significantly upregulated in both mRNA sequencing and Nanostring analysis. One inflammation-related gene, EGR1, was significantly downregulated in both techniques. In RNA sequencing only, 11 inflammation-related genes were significantly upregulated. Four inflammation-related genes were significantly upregulated in the Nanostring analysis only—C1QB, TLR2, S100A9 and FKBP5. In RNA sequencing only, five inflammation-related genes were significantly downregulated. In Nanostring analysis only, one inflammation-related gene was significantly downregulated—GPR85 (Table 4). One GO term was significantly upregulated, “positive regulation of oligodendrocyte progenitor proliferation” (Fig. 3), aligning with motor cohort EWCE results.

**Table 4:** Inflammation-related differential gene expression in Huntington’s disease motor cases in mRNA sequencing and Nanostring analyses

Motor HD significantly upregulated inflammation-related genes					
Gene name	Description	RNA sequencing		Nanostring	
		Adj *P* value	Log2 fold change	Adj *P* value	Log2 fold change
SERPINA5	Serpin family A member 5	0.0004***	3.7144	0.0224*	1.9564
NGFR	Nerve growth factor receptor	0.0005***	3.0443	0.0458*	1.5256
CD44	CD44 molecule	0.0039**	2.7435	0.0106*	2.1132
GJC2	Gap junction protein gamma 2	0.0067**	1.4092	0.0087**	0.9244
SPP1	Secreted phosphoprotein 1	0.0243*	1.9629	0.0217*	1.3791
AQP4	Aquaporin 4	0.0485*	1.4249	0.0032**	0.8867
SIGLEC8	Sialic acid binding Ig like lectin 8	0.0546*	1.5804	0.0222*	0.9112
*Statistical significance only in RNA sequencing*		*Adj P value*		*Log2 fold change*	
C4B	Complement C4B	0.0001***		3.0350	
NTS	Neurotensin	0.0003***		4.7794	
SLC5A11	Solute carrier family 5 member 11	0.0033**		2.1656	
CHI3L1	Chitinase 3-like 1	0.0038**		1.3892	
P2RX7	Purinergic receptor P2X 7	0.0175**		1.3976	
TNC	Tenascin C	0.0179**		2.4474	
ALOX5AP	Arachidonate 5-lipoxygenase activating protein	0.0203*		2.2377	
CYP4F3	Cytochrome P450 family 4 subfamily F member 3	0.0269*		1.7332	
RNASE2	Ribonuclease A family member 2	0.0304*		2.5265	
C3	Complement C3	0.0454*		1.6652	
CD163	CD163 molecule	0.0490*		2.7601	
*Statistical significance only in NanoString*		*Adj P value*		*Log2 fold change*	
S100A9	S100 calcium-binding protein A9	0.0123*		2.7092	
FKBP5	FK506 binding protein 5	0.0337*		1.2871	
C1QB	Complement C1q B chain	0.0512*		1.1043	
TLR2	Toll-like receptor 2	0.0541*		1.2359	
**Motor HD significantly downregulated inflammation-related genes**					
Gene name	Description	RNA sequencing		Nanostring	
		Adj *P* value	Log2 fold change	Adj *P* value	Log2 fold change
EGR1	Early growth response 1	<0.0001*	−1.4197	0.0188*	−0.9743
*Statistical significance only in RNA sequencing*		*Adj P value*		*Log2 fold change*	
TDGF1	Teratocarcinoma-derived growth factor 1	0.0107*		−2.6020	
EGR2	Early growth receptor 2	0.0236*		−1.4368	
UTS2B	Urotensin 2B	0.0288*		−1.9629	
GRP	Gastrin-releasing peptide	0.0413*		−1.6061	
CCL8	C-C motif chemokine ligand 8	0.0541*		−5.2818	
*Statistical significance only in NanoString*					
GPR85	G protein-coupled receptor 85	0.0025**		−0.5952	

RNA sequencing—two-tailed Wald test; Nanostring—two-tailed Mann–Whitney test for HD vs control, and a right-tailed Kruskal–Wallis test to compare the control vs HD symptom groups. * *p* ≤ 0.05; ** *p* ≤ 0.01; *** *p* ≤ 0.001.

### Inflammation-related differential gene expression in mixed Huntington’s disease cases

Mixed HD cases showed no overlapping upregulated inflammation-related genes for RNA sequencing and Nanostring analysis (Table 5). Two genes, TNFSF10 and CD8B2, was significantly downregulated in both RNA sequencing and Nanostring in HD mixed cases. In Nanostring analysis only, multiple genes encoding for chemokines and EGR1 were downregulated, whereas in mRNA sequencing only VCAM1 was downregulated (Table 5). Two inflammation-related GO terms were significantly upregulated for HD mixed cases—“alpha9-beta1 integrin- vascular cell adhesion molecule-1 complex” and “TRAIL binding.”

**Table 5:** Inflammation-related differential gene expression in Huntington’s disease mixed cases in mRNA sequencing and Nanostring analyses

Mixed HD significantly upregulated inflammation-related genes					
Gene name	Description	Adj *P* value		Log2 fold change	
Statistical significance only in RNA sequencing					
SERPINA5	Serpin family A member 5	0.0519*		2.6752	
C4B	CD244 molecule	0.0261*		2.5610	
*Statistical significance only in Nanostring*					
CHI3L1	Chitinase 3-like 1	0.0015**		2.1461	
TMIGD3	Transmembrane and immunoglobulin domain-containing 3	0.0109*		1.0166	
S100A9	S100 calcium-binding protein A9	0.0376*		2.5774	
SIGLEC8	Sialic acid binding Ig like lectin 8	0.0429*		0.7001	
**Mixed HD significantly downregulated inflammation-related genes**					
Gene name	Description	RNA sequencing		Nanostring	
		Adj *P* value	Log2 fold change	Adj *P* value	Log2 fold change
CD8B2	CD8b2 molecule	0.0214*	−2.4246	0.0170*	−1.4503
TNFSF10	TNF superfamily member 10	0.0221*	−2.0943	0.0504*	−1.3152
*Statistical significance only in RNA sequencing*		*Adj P value*		*Log2 fold change*	
VCAM1	Vascular cell adhesion molecule 1	0.0120*		−1.9659	
*Statistical significance only in Nanostring*		*Adj P value*		*Log2 fold change*	
GPR85	G protein-coupled receptor 85	0.0164*		−0.4693	
EGR1	Early growth response 1	0.0422*		−0.9011	
CCL3L1	C-C motif chemokine ligand 3-like 1	0.0082*		−2.4828	
CCL4L2	C-C motif chemokine ligand 4 like 2	0.0283*		−1.5327	
CXCL12	C-X-C motif chemokine ligand 12	0.0170*		−0.7467	

RNA sequencing—two-tailed Wald test; Nanostring—two-tailed Mann–Whitney test for HD vs control, and a right-tailed Kruskal–Wallis test to compare the control vs HD symptom groups. * *p* ≤ 0.05; ** *p* ≤ 0.01.

### Stereological estimates of the MCC in control and Huntington’s disease

**Fig. 4:**
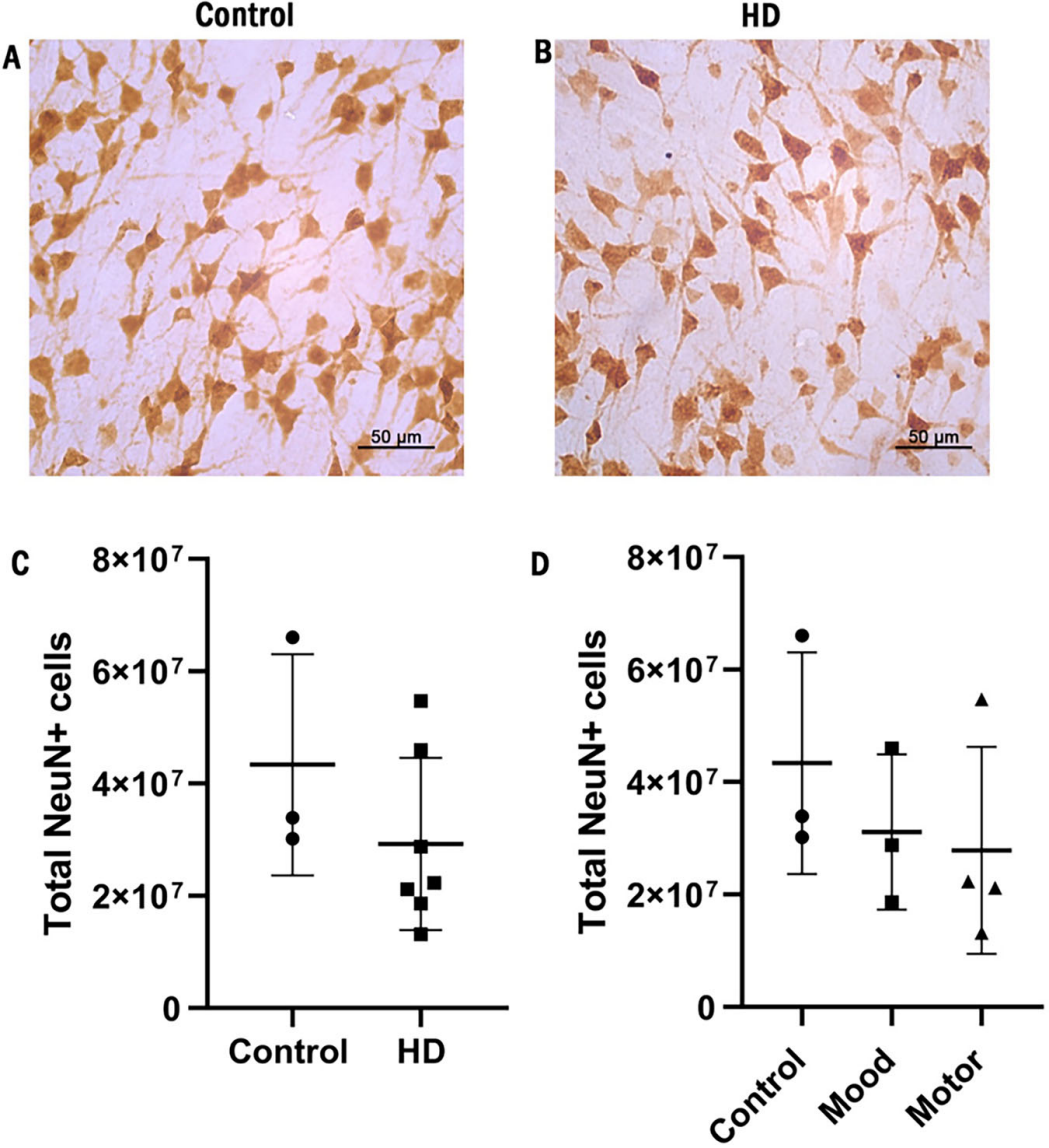
Total NeuN-positive neurons estimated in the middle cingulate gyrus in Huntington’s disease and control cases. **A** NeuN immunolabeling in control middle cingulate cortex (MCC). **B** NeuN immunolabeling in Huntington’s disease MCC. **C** Stereological estimations of total NeuN-positive neuron cell number in controls and all HD cases. Two-tailed Mann–Whitney *U* test, control *n* = 3, HD *n* = 7. **D** Stereological estimation of the total NeuN-positive neuron cell number between controls, the mood symptom group, and the motor symptom group. Right-tailed Kruskal–Wallis test, Control *n* = 3, Mood *n* = 3, Motor *n* = 4. Scale bar: **A**, **B:** 50 µm. Data are presented as mean ± SD.

NeuN immunoreactivity was present in the cell bodies and proximal processes of neurons in the MCC (Fig. 4A, B). Stereological sampling methods were employed to estimate the number of NeuN-positive neurons in the MCC in control and HD cases. Analysis of the stereological estimates of the total NeuN-positive neuron number in the MCC showed no significant changes in cell number between control cases and pooled HD cases (Fig. 4C). No significant differences in total NeuN-positive cell number were detected between mood and motor symptom groups and in these cases compared to controls when symptom-specific subgrouping was applied (Fig. 4D).

## Discussion

This study reports inflammation-related differential gene expression in the HD MCC using whole transcriptome RNA sequencing and NanoString nCounter assay validation. Our RNA sequencing identified 223 differentially expressed genes, 37 of which were inflammation-related—24 upregulated and 13 downregulated. Our NanoString nCounter analysis validated 19 upregulated and nine downregulated of these inflammation-related genes, and identified an additional four upregulated and two downregulated genes. TLRs, classical complement, and other genes, such as AQP4, were upregulated in the HD MCC. However, chemokine genes were downregulated, and glial markers and cytokines were not differentially expressed. RELA and STAT3 were upregulated only when using NanoString nCounter analysis, and the cell-type enrichment analysis suggested that activated microglia were significantly enriched within the HD MCC.

Many of the upregulated genes reported in this study have pre-established neuroinflammatory roles. The “inflammatory response” GO term was significantly upregulated across all HD cases based on 16 significantly upregulated genes, suggesting the involvement of multiple inflammatory processes in the MCC. Multiple classical complement genes, including C1Qa, C1Qb, C3, C3AR1, and C4B, were upregulated in the HD MCC. Previous studies show increased classical complement protein expression in the HD striatum, motor cortex, caudate nucleus, and CSF, along with complement gene upregulation in the caudate nucleus^23–26^. mHTT likely activates classical complement after striatal neuronal damage in early HD^27^. Although our cases represent later HD stages, complement activation possibly occurs later in the MCC, as disease pathology likely affects it later. Our PPI analysis suggested that complement genes contribute to specific inflammatory processes. The “synaptic pruning” and “neuron remodeling” GO terms were significantly enriched, involving C3, C1QA, and C1QB. Previous studies suggest that classical complement stimulates microglial-mediated synaptic pruning in HD—therefore, upregulation could lead to the removal of healthy synapses, dysfunctional remodeling, and subsequent functional impairment^11,28,29^. C1QA and C1QB are associated with corticostriatal synapse loss in HD^30^, and excessive C1Q-dependent synaptic pruning is thought to occur in AD and demyelinating diseases^31–33^. Similarly, PPI analysis of the upregulated classical complement genes showed significant enrichment of apoptotic cell clearance and opsonization.

TLR 2, 7, and 8 genes were upregulated in HD MCC, as was the GO term “toll-like receptor binding.” TLRs can provoke cytokine secretion, gliosis, and neuronal apoptosis alongside myeloid differentiation primary-response protein 88 (MyD88), S100A9, and P2RX7^34–38^. TLRs are likely implicated in HD neuroinflammatory responses, with TLR2 deficiency increasing HD mice’s lifespan^27,39^. “I$\kappa$B phosphorylation” was significantly enriched in PPI analysis involving TLR2 and TLR7. I$\kappa$B phosphorylation releases NF-$\kappa$B, a family of critical transcription factors that enable pro-inflammatory gene upregulation, glial activation, and cytokine production^40^. NF-$\kappa$B may be involved in HD pathophysiology, given mHTT’s interaction with IKK and NF-$\kappa$B release in PC12 cell cultures, astrocytic NF-$\kappa$B nuclear localization, and pro-inflammatory cytokine efflux in the human HD caudate nucleus, as well as increased NF-$\kappa$B activity in mHTT expressing microglia stimulated by IL-6^41,42^. RELA, an NF-$\kappa$B subunit, was upregulated in the Nanostring analysis but not in RNA sequencing. “Positive regulation of cytokine production” was also significantly enriched in PPI analysis involving TLRs. Specific pro-inflammatory cytokines included interleukin (IL) 1$\beta ,\,$IL-6, IL-8, and interferon (IF) $\alpha ,\,\beta ,$ and $\gamma$. Cytokines mediate inflammatory responses, promoting STAT3, NF-$\kappa$B, and glial activation and potentially mediating chronic neuroinflammation and neuron loss in HD^11^. Increased cortical IL-6 and IL-8, striatal IL-6, IL-8, and IF-α and plasma IL-1β, IL-6, and IL-8 are seen in HD^43^. Higher plasma IL-6 and IF-α correlate with worsened depression in HD, and plasma IL-6 levels increase with HD severity^44,45^. However, we did not observe cytokine upregulation, implying that their levels do not change in the HD MCC.

Pro-inflammatory chemokine genes CCL4, CCL8, CCL3L1, CCL4L2, and CXCL12 were significantly downregulated across all HD cases in the RNA sequencing and Nanostring analyses. The chemokine-related GO and PPI analysis indicated significant de-enrichment of monocyte, eosinophil, and leukocyte chemotaxis, as well as CCR chemokine receptor binding, chemokine-mediated signaling pathways, and cellular response to IL-1, TNF-$\alpha$, and IF-$\gamma$. These results are unexpected as chemokines are elevated in the human HD striatum, and CCL5 is elevated in the human HD frontal cortex, caudate, and substantia niagra^26^. Chemokines are cytokines primarily known for mediating immune cell chemotaxis, but their other roles include immune cell development, differentiation, and proliferation, cytokine production, and cell arrest^46^. Their downregulation in the MCC suggests potential impairment of these roles, with a correspondingly skewed inflammatory response. Chemokine downregulation may be a compensatory mechanism to minimize neuroinflammation, particularly given that overt neuroinflammatory markers such as glial markers and pro-inflammatory cytokines are not upregulated in the HD MCC. De-enriched cellular response to IL-1, TNF-$\alpha ,$ and IF-$\gamma$ in the PPI analysis supports this and may counteract the upregulation of IF-$\alpha$, IF-$\beta$, IF-$\gamma$, IL-1$\beta$, IL-6, and IL-8 in the TLR PPI analysis. Downregulated chemokine transcripts may also be a sign of glial dysfunction, given that glia express and secrete chemokines. Glial dysfunction in the MCC is suggested in a previous study from our laboratory^15^, and is supported by enriched activated microglia in the MCC within this study’s EWCE analysis. However, further studies are required to test this proposed hypothesis.

The EWCE analysis indicated that two of the four cell types enriched in the HD MCC were activated microglia—MGL2 and MGL3. This aligns with our previous study in the HD MCC, which found that while there was no microglial proliferation, the microglia present were predominantly activated morphologies, such as rod, hypertrophic, and dystrophic microglia. Ameboid microglia were reduced, potentially suggesting a reduced ability to clear mHTT, as ameboid microglia mediate phagocytosis^15^. Upregulated phagocytosis-related processes in this study’s PPI analysis, such as opsonization, may reflect attempted compensation. Five oligodendrocyte cell types were enriched in the HD motor cohort—MOL2 (mature oligodendrocytes), MFOL1 and MFOL2 (myelin-forming oligodendrocytes), and NFOL1 and NFOL2 (newly formed oligodendrocytes). Oligodendrocytes are implicated in neuroinflammation, producing and expressing receptors for pro-inflammatory cytokines^47^. Oligodendrocytes undergo developmental impairment in HD, suggesting their roles are compromised^48^.

Inflammation-related differential gene expression was present in the mood, motor, and mixed HD cohorts, each with a unique set of differentially expressed genes. However, PPI analysis of the mood and mixed gene sets indicated no protein interactions, and EWCE analysis found no enriched cells. These findings were unexpected as a previous study from our laboratory indicated that HD mood cases have more activated microglia morphologies than controls. However, microglia do not reach HLA-DP/DQ/DR immunoreactivity, have reduced ameboid microglial morphologies, and astrocytes present reduced EAAT2 immunoreactivity in the MCC^15^. While the MCC projects to mood-related regions, such as the prefrontal cortex and limbic structures, it also projects to the premotor and motor cortices and the basal ganglia^13,49^. HD motor cases had more differentially expressed inflammation-related genes than mood and mixed cases, with eight validated differentially expressed genes and 24 overall. AQP4 was upregulated in HD motor cases, which has a significant role in neuroinflammatory responses^50^. HD motor PPI analysis indicated multiple interactions between differentially expressed proteins. EWCE analysis also suggested oligodendrocyte enrichment in HD motor cases, which can be involved in neuroinflammation^46^. These findings suggest that the neuroinflammatory environment in the motor HD MCC may be distinct and warrant further investigation to determine if it contributes to symptomatology.

The intricate dysregulation in the expression of genes related to inflammatory processes suggests the possibility of a complex pathological inflammatory profile within the MCC in HD. Pro-inflammatory processes, including microglial activation, have been postulated to underlie neuronal death^9^. Interestingly, when a stereological analysis of cell number was carried out, the MCC remained robust to cellular degeneration between HD and control cases. This was sustained when symptom subgrouping was compared. We hypothesize that while pathological alterations in gene expression may indicate changes in the inflammatory state of the HD MCC, inflammatory cells are primed for inflammatory processes but may not reach a state of classical activation. Previous research conducted by our laboratory supports this hypothesis^15^. Immunohistochemical studies of the MCC revealed morphological changes in microglia in HD cases that are indicative of a pro-inflammatory role, but no change in overall HLA-DP, DQ, DR expression, a marker of classic microglial activation. These study findings suggested that microglia did not present protein expression patterns associated with classical activation in the HD MCC^15^. Furthermore, as found in this current study, a lack of gene expression alterations in classical activation markers, such as pro-inflammatory cytokines, also supports this hypothesis. It is possible that a lack of classical activation of inflammatory cells may be one contributing factor to the lack of cell death in the HD MCC.

We acknowledge that a limitation of this work is a relatively low sample size for a transcriptomic study. The sparse number of cases investigated in this study was due to a lack of availability of tissue samples after applying the strict case selection criteria to reduce variability related to case characteristics and sample processing. However, this study provides seminal data about the complexity of inflammatory gene expression changes in the MCC, which is now established, and can be the focus of further research. As a next step, further validation of the results is required. RNAseq has a relatively high false discovery rate, and Nanostrating analysis has limitations in reliably detecting transcripts with low expression, which most likely contributed to discrepancies reported in this study. Therefore, all relevant DEGs will have to be examined at the protein level, and also their spatial, cell-type-specific distribution. Further in vivo animal studies will be required to understand the functional relevance of the observed DEGs and the impact on HD pathology. Because the MCC may have a unique role in motor symptomatology, a much larger sample size and data from different patient cohorts will be required to validate our findings and determine if the observed gene signature in the HD MCC serves as a biomarker or a mechanistic driver of symptom heterogeneity.

This study is the first to investigate neuroinflammatory differential gene expression in the HD MCC. While overt neuroinflammatory markers, such as pro-inflammatory cytokines, are not altered, a complex mix of inflammation-related processes is occurring. Upregulation of classical complement and TLRs may be causing a dysfunctional “gain in function” in processes such as synaptic pruning and neuron remodeling, yet the downregulation of chemokines is likely leading to “loss of function” in processes such as immune cell chemotaxis. There may also be compensatory mechanisms at play involving cytokine production and response, but further research is required to explore these. Motor HD cases show some characteristic pathology, such as oligodendrocyte cell enrichment, but it is the only symptom group with inflammatory protein interactions. This suggests that the MCC may have a unique role in motor symptomatology.

## Supplementary information

Supplementary Information

## Data Availability

Data are available in the main manuscript, the supplementary material, and the source data for Figs. 1–3 at the University of Auckland Figshare data repository (https://figshare.com/s/5135bc8d4dd9e2788bbd). The EWCE analysis source data are provided in Supplementary Table 3. The data related to up- and down-regulated Gene Ontology terms are presented in Supplementary Tables 5–9.
