# Supplementary material for: Exploration of the genetic neuroinflammatory environment in the human midcingulate cortex in Huntington’s disease
Source: Commun Med (Lond). 2026 Jun 4;6:324. doi: 10.1038/s43856-026-01526-5 (PMC13237170; doi:10.1038/s43856-026-01526-5)
Supplement: Supplementary file 1 — Supplementary Information [file 43856_2026_1526_MOESM1_ESM.pdf]

Supplementary Information

Supplementary Table 1. **Post-mortem human Huntington's disease cases used in this study, including RNA sequencing and Nanostring experiments. An asterisk indicates cases used only for RNA sequencing.**

Case number	HD grade	Age (years)	Sex	Post-mortem delay (PMD) (hours)	CAG repeat number	Cause of death	Brain weight (g)
**Motor cases**							
HC072*	HD2	60-65	F	24	17/42	Pneumonia	1070
HC075	HD3	40-45	M	5.5	18/52	Pneumonia	1085
HC087	HD4	45-50	M	18	20/49	Bronchopneumonia	950
HC110	HD4	40-45	M	29	20/51	Aspiration pneumonia	1147
**Mood cases**							
HC077	HD4	50-55	F	9	17/53	Pneumonia	1010
HC085	HD3	60-65	F	19	24/44	Unknown	1160
HC092	HD1	70-75	M	5	17/41	Pneumonia	1190
HC148	HD3	60-65	M	16	22/43	Pulmonary embolism	1226
HC149	HD2	50-55	M	6.5	20/39	Pneumonia	1272
**Mixed cases**							
HC079	HD1	55-60	F	4	17/42	Cardiorespiratory failure	1190
HD084	HD3	40-45	F	3.5	21/49	Acute renal failure	970
HC114	HD2	50-55	F	12	21/47	Pneumonia	961
HC119	HD3	50-55	M	15.5	17/48	Dehydration	1007
HC133	HD2	60-65	M	14	17/43	Renal failure	1224

Supplementary Table 2. Post-mortem human control cases used in this study, including RNA sequencing and Nanostring experiments.

Case number	Age (years)	Sex	Post-mortem delay (PMD)	Cause of death	Brain weight (g)
H132	60-65	F	12	Ruptured aorta	1280
H159	50-55	M	16.5	Ischemic Heart Disease	1215
H168	60-65	M	9	Ischemic Heart Disease	1432
H170	60-65	M	17	Ischemic Heart Disease	1370
H190	70-75	F	19	Ruptured myocardial infarction	1264
H204	65-70	M	9	Ischemic Heart Disease	1461
H238	60-65	F	16	Dissecting aortic aneurysm	1324
H157	65-70	M	15	Ischemic Heart Disease	1360
H239	60-65	M	15.5	Ischemic Heart Disease	1529

Supplementary Table 3: EWCE analysis of all HD, motor HD, mood HD and mixed HD differential gene expression from mRNA sequencing results.

Set	Total DEGs	Genes after filtering	Cell Types q < 0.05	Cell Type	p-value	Fold Change	q -value
HD_all	223	82	4	MGL3	4.00E-05	4.0362	0.0106
				TEGLU17	1.00E-04	5.3117	0.0133
				VECA	0.00022	2.7211	0.0179
				MGL2	0.00027	2.8482	0.0179
HD_Motor	193	99	11	MOL2	0	3.5485	0
				TEGLU17	0.00011	4.1488	0.0115
				MEINH5	0.00013	4.2021	0.0115
				MFOL1	0.00018	2.7299	0.0119
				NFOL1	0.00033	2.2320	0.0175
				MFOL2	0.00071	2.2755	0.0314
				ENTG3	0.00097	2.7228	0.0354
				NFOL2	0.00107	2.0977	0.0354
				SCINH5	0.00124	1.8718	0.0365
				SCGLU10	0.00146	2.4364	0.0387
				HYPEP2	0.00177	2.5661	0.0426
HD_Mood	29	11	0				
HD_Mixed	20	9	0				

Notes: One-tailed bootstrap hypothesis test

Supplementary Table 4: Inflammatory-related gene probe design for NanoString mRNA analysis. Housekeeping genes are indicated with an asterisk.

ACTB *	NM_001101.3	GJC2	NM_020435.2
B2M *	NM_004048.2	GPR85	NM_001146267.1
G6PD *	NM_000402.4	GRP	NM_001012513.1
POLR1B *	NM_019014.4	IL17RB	NM_018725.3
GAPDH *	NM_001256799.1	NGFR	NM_002507.3
TOP1 *	NM_003286.2	NTS	NM_006183.4
ALOX5AP	NM_001629.2	P2RX7	NM_002562.5
AQP4	NM_004028.3	RELA	NM_021975.3
C1QA	NM_015991.2	RNASE2	NM_002934.2
C1QB	NM_000491.3	S100A9	NM_002965.3
C3	NM_000064.2	SEMA3G	NM_020163.1
C3AR1	NM_004054.3	SERPINA1	NM_000295.4
C4B	NM_001002029.3	SERPINA3	NM_001085.4
CCL3L1	NM_021006.4	SERPINA5	NM_000624.4
CCL4	NM_002984.2	SIGLEC8	NM_014442.2
CCL4L2	NM_001291468.1	SLC5A11	NM_001258411.1
CCL8	NM_005623.2	SPP1	NM_000582.2
CD163	NM_004244.4	STAT3	NM_003150.3
CD244	NM_001166663.1	TDGF1	NM_003212.2
CD44	NM_001001392.1	TLR2	NM_003264.3
CD8B2	NM_001349727.1	TLR7	NM_016562.3
CHI3L1	NM_001276.2	TLR8	NM_016610.2
CXCL12	NM_199168.3	TMIGD3	NM_001081976.1
CXCR4	NM_003467.2	TNC	NM_002160.3
EGR1	NM_001964.2	TNFSF10	NM_003810.3
EGR2	NM_000399.3	UTS2B	NM_198152.3
EVI2B	NM_006495.3	VCAM1	NM_001078.3
FKBP5	NM_001145775.1		

Supplementary Table 5. Upregulated inflammation-related Gene Ontology terms across all Huntington's disease cases.

GO enrichment pathway name	GO enrichment pathway ID	P-value	Adjusted p-value	Number of genes linked to GO term with significant differential expression	Genes linked to GO term with significant differential expression
**Cellular Components**					
Macrophage migration inhibitory factor receptor complex	GO:0035692	0.00756	0.09144	1	CD44
Toll-like receptor 1-Toll-like receptor 2 protein complex	GO:0035354	0.00756	0.09144	1	TLR2
Complement component C1 complex	GO:0005602	0.00756	0.09144	1	C1QB
**Molecular Function**					
Arachidonic acid binding	GO:0050544	0.00015	0.01346	2	ALOX5AP, S100A9
Toll-like receptor binding	GO:0035325	0.001	0.05983	2	TLR2, S100A9
C5L2 anaphylatoxin chemotactic receptor binding	GO:0031715	0.00397	0.10565	1	C3
Complement component C3a receptor activity	GO:0004876	0.00397	0.10565	1	C3AR1
Arachidonate 5-lipoxygenase activity	GO:0004051	0.01187	0.14694	1	ALOX5AP
Arachidonate 15-lipoxygenase activity	GO:0050473	0.01187	0.14694	1	ALOX5AP
**Biological Process**					
Inflammatory response	GO:0006954	0.00023	0.04311	16	SLC7A2, CD44, P2RX7, SPP1, TMIGD3, C3, ADGRE2, ALOX5AP, CHI3L1, TLR2, MMP3, S100A9, C3AR1, SERPINA3, SERPINA1, C4B
Acute inflammatory response	GO:0002526	0.00151	0.17262	4	C3, ALOX5AP, SERPINA3, SERPINA1
Cellular response to nitric oxide	GO:0071732	0.00186	0.18712	2	CCNA2, MMP3
Neutrophil degranulation	GO:0043312	0.00219	0.19540	8	CD44, C3, CHI3L1, TLR2, S100A9, C3AR1, SERPINA3, SERPINA1
Positive regulation of apoptotic cell clearance	GO:2000427	0.00263	0.19534	2	C3, C4B

Notes: Two-tailed Fisher's exact test.

Supplementary Table 6. Downregulated inflammation-related Gene Ontology terms across all Huntington's disease cases.

GO enrichment pathway name	GO enrichment pathway ID	P-value	Adjusted p-value	Number of genes linked to GO term with significant differential expression	Genes linked to GO term with significant differential expression
**Molecular Function**					
Chemokine activity	GO:0008009	0.0000001	0.000022	5	CXCL12, CCL8, CCL4, CCL4L2, CCL3L1
CCR chemokine receptor binding	GO:0048020	0.0000056	0.000616	4	CCL8, CCL4, CCL4L2, CCL3L1
TRAIL binding	GO:0045569	0.0089	0.150615	1	TNFSF10
CXCR chemokine receptor binding	GO:0045236	0.0194	0.185565	1	CXCL12
CCR1 chemokine receptor binding	GO:0031726	0.0194	0.185565	1	CCL4
CCR5 chemokine receptor binding	GO:0031730	0.0212	0.194333	1	CCL4
**Biological Process**					
Monocyte chemotaxis	GO:0002548	0.00000037	0.000497	5	CXCL12, CCL8, CCL4, CCL4L2, CCL3L1
Chemokine mediated signalling pathway	GO:0070098	0.000021	0.005641	5	CXCL12, CCL8, CCL4, CCL4L2, CCL3L1
Lymphocyte chemotaxis	GO:0048247	0.0000094	0.004208	4	CCL8, CCL4, CCL4L2, CCL3L1
Neutrophil chemotaxis	GO:0030593	0.000048	0.010744	4	CCL8, CCL4, CCL4L2, CCL3L1
Cellular response to interleukin-1	GO:0071347	0.00015	0.028779	4	CCL8, CCL4, CCL4L2, CCL3L1
Eosinophil chemotaxis	GO:0048245	0.00002	0.005641	3	CCL8, CCL4, CCL4L2
Positive regulation of ERK1 and ERK2 cascade	GO:0070374	0.00083	0.105202	4	CCL8, CCL4, CCL4L2, CCL3L1
Cellular response to tumour necrosis factor	GO:0071356	0.0014	0.109998	4	CCL8, CCL4, CCL4L2, CCL3L1
Cellular response to interferon-gamma	GO:0071346	0.00142	0.109998	4	CCL8, CCL4, CCL4L2, CCL3L1
Positive regulation of chemotaxis	GO:0050921	0.00202	0.12331	3	CXCL12, SMOC2, CCL4
Positive regulation of lymphocyte migration	GO:2000403	0.00307	0.15355	2	CXCL12, CCL4

Notes: Two-tailed Fisher's exact test.

Supplementary Table 7. Inflammation-related Gene Ontology terms in Huntington's disease mood cases compared to control cases.

Upregulated or downregulated	GO enrichment pathway name	GO enrichment pathway ID	P-value	Adjusted p-value	Number of genes linked to GO term with significant differential expression	Genes linked to GO term with significant differential expression
**Molecular Function**						
Upregulated	Arachidonate 5-lipoxygenase activity	GO:0004051	0.00153	0.05922	1	ALOX5AP
Upregulated	Arachidonic acid binding	GO:0050544	0.00254	0.05922	1	ALOX5AP
Upregulated	Leukotriene-C4 synthase activity	GO:0004464	0.00254	0.05922	1	ALOX5AP
Upregulated	Interleukin-17 receptor activity	GO:0030368	0.00407	0.05922	1	IL17RB
Upregulated	Glutathione peroxidase activity	GO:0004602	0.01216	0.12639	1	ALOX5AP
Upregulated	Complement binding	GO:0001848	0.01618	0.12944	1	C4B
Upregulated	Oxidoreductase activity, acting on peroxide as an acceptor	GO:0016684	0.03114	0.15853	1	ALOX5AP
Upregulated	Antioxidant activity	GO:0016209	0.04881	0.202471	1	ALOX5AP
**Biological Process**						
Downregulated	Negative regulation of lymphocyte migration	GO:2000402	0.00353	0.17238	1	ADTRP
Upregulated	Inflammatory response	GO:0006954	0.00244	0.19759	5	IL17RB, TMIGD3, ALOX5AP, CHI3L1, C4B
Upregulated	Leukotriene production involved in inflammatory response	GO:0002540	0.00166	0.19759	1	ALOX5AP
Upregulated	Positive regulation of inflammatory response	GO:0050729	0.00423	0.22882	2	IL17RB, ALOX5AP
Upregulated	Interleukin-17 mediated signalling pathway	GO:0097400	0.00499	0.22882	1	IL17RB
Upregulated	Activation of NF-kappaB inducing kinase activity	GO:0007250	0.00995	0.29309	1	CHI3L1
Upregulated	Positive regulation of apoptotic cell clearance	GO:2000427	0.0105	0.29309	1	C4B
Upregulated	Leukotriene biosynthetic process	GO:0019370	0.01105	0.29558	1	ALOX5AP
Upregulated	Regulation of apoptotic cell clearance	GO:2000425	0.0116	0.29789	1	C4B
Downregulated	Negative regulation of leukocyte cell-cell adhesion	GO:1903038	0.03826	0.42583	1	ADTRP

Notes: Two-tailed Fisher's exact test.

Supplementary Table 8. Inflammation-related Gene Ontology terms in Huntington's disease motor cases compared to control cases.

Upregulated or downregulated	GO enrichment pathway name	GO enrichment pathway ID	P-value	Adjusted p-value	Number of genes linked to GO term with significant differential expression	Genes linked to GO term with significant differential expression
**Cellular Components**						
Upregulated	Macrophage migration inhibitory factor receptor complex	GO:0035692	0.00945	0.12843	1	CD44
**Molecular Function**						
Upregulated	C5L2 anaphylatoxin chemotactic receptor binding	GO:0031715	0.005	0.17038	1	C3
Upregulated	20-aldehyde-leukotriene B4 20-monooxygenase activity	GO:0097259	0.0101	0.20337	1	CYP4F3
Upregulated	20-hydroxy-leukotriene B4 omega oxidase activity	GO:0097258	0.0101	0.20338	1	CYP4F3
Upregulated	Arachidonate 5-lipoxygenase activity	GO:0004051	0.015	0.2215	1	ALOX5AP
**Biological Process**						
Upregulated	Positive regulation of oligodendrocyte progenitor proliferation	GO:0070447	0.000074	0.01961	2	LRP2, GJC2
Upregulated	Positive regulation of apoptotic cell clearance	GO:2000427	0.004	0.22466	2	C3, C4B
Downregulated	Cellular response to interleukin-8	GO:0098759	0.00302	0.26603	1	EGR1
Downregulated	Cellular response to mycophenolic acid	GO:0071506	0.00202	0.26603	1	EGR1

Notes: Two-tailed Fisher's exact test.

Supplementary Table 9. Inflammation-related Gene Ontology terms in Huntington's disease mixed cases compared to control cases.

Upregulated or downregulated	GO enrichment pathway name	GO enrichment pathway ID	P-value	Adjusted p-value	Number of genes linked to GO term with significant differential expression	Genes linked to GO term with significant differential expression
**Cellular Components**						
Downregulated	Alpha9-beta1 integrin-vascular cell adhesion molecule-1 complex	GO:0071065	0.00047	0.03008	1	VCAM1
**Molecular Function**						
Downregulated	TRAIL binding	GO:0045569	0.00102	0.01049	1	TNFSF10
Downregulated	Tumour necrosis factor receptor binding	GO:0005164	0.01055	0.07560	1	TNFSF10
Downregulated	Tumour necrosis factor receptor superfamily binding	GO:0032813	0.01338	0.08219	1	TNFSF10
**Biological Process**						
Downregulated	Chronic inflammatory processes	GO:0002544	0.0052	0.18349	1	VCAM1
Downregulated	Leukocyte tethering or rolling	GO:0050901	0.0062	0.18349	1	VCAM1
Downregulated	Positive regulation of release of cytochrome c from mitochondria	GO:0090200	0.0066	0.18349	1	TNFSF10
Downregulated	Negative regulation of extrinsic apoptotic signaling pathway via death domain receptors	GO:1902042	0.0074	0.18349	1	TNFSF10
Downregulated	Positive regulation of extrinsic apoptotic signaling pathway	GO:2001238	0.0101	0.21219	1	TNFSF10
Downregulated	Activation of cysteine-type endopeptidase activity involved in apoptotic process	GO:0006919	0.0192	0.27297	1	TNFSF10
Downregulated	Acute inflammatory response	GO:0002526	0.0244	0.27297	1	VCAM1
Downregulated	B cell differentiation	GO:0030183	0.0258	0.27297	1	VCAM1
Downregulated	Positive regulation of T cell proliferation	GO:0042102	0.0276	0.27297	1	VCAM1

Notes: Two-tailed Fisher's exact test.

Supplementary Figure 1: Inflammation-related protein-protein interaction analysis of all HD motor and mixed cases.

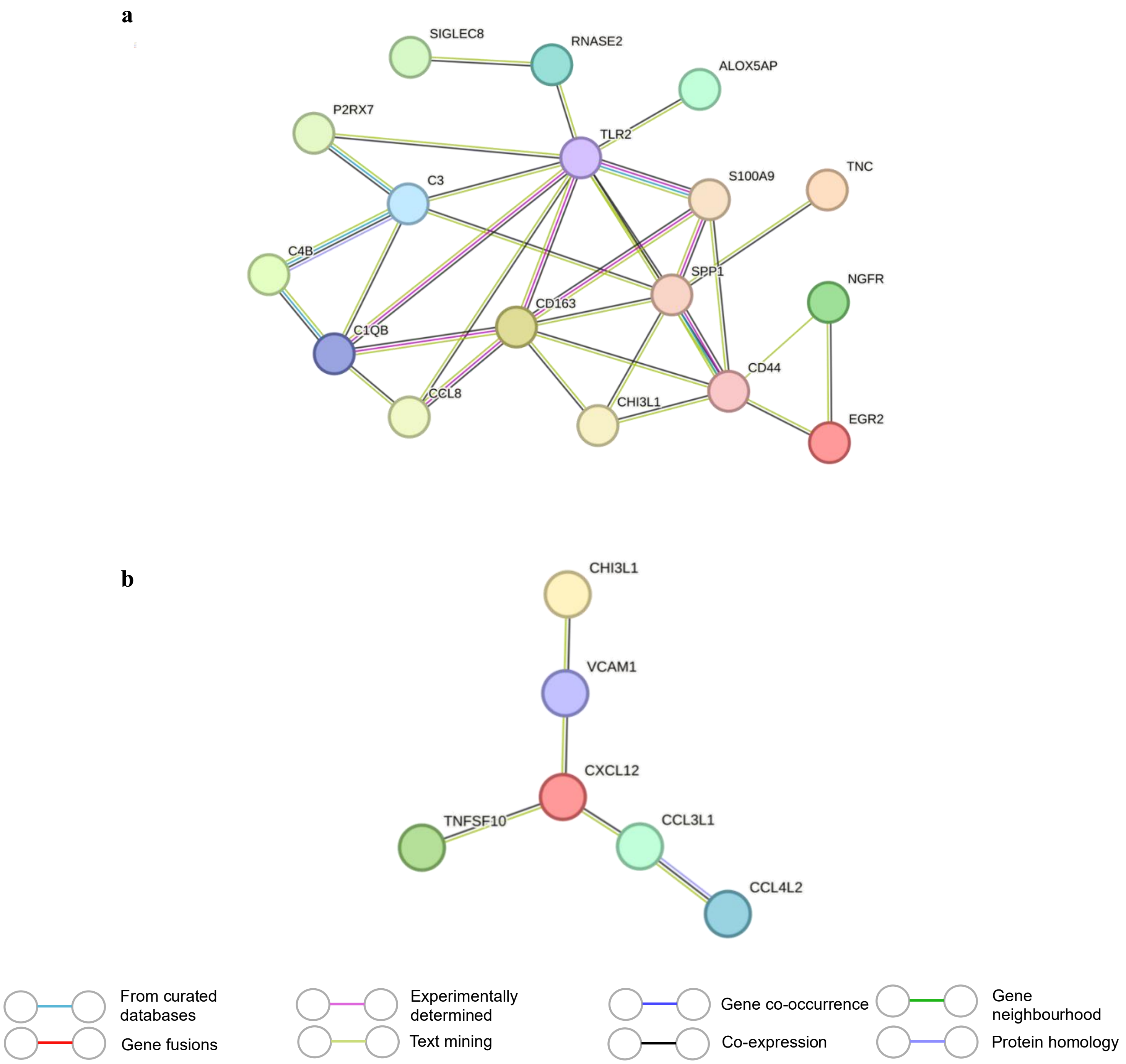

a, Protein-protein interaction analysis for all inflammation-related differentially expressed genes in the MCC for the HD motor cohort. **b,** Protein-protein interaction analysis for all inflammation-related differentially expressed genes in the MCC for the HD mixed cohort.
